# The MTB/MDR ELITe MGB^®^ Kit: Performance Assessment for Pulmonary, Extra-Pulmonary, and Resistant Tuberculosis Diagnosis, and Integration in the Laboratory Workflow of a French Center

**DOI:** 10.3390/pathogens10020176

**Published:** 2021-02-06

**Authors:** Elisabeth Hodille, Charlotte Genestet, Thomas Delque, Luna Ruffel, Yvonne Benito, Isabelle Fredenucci, Jean-Philippe Rasigade, Gérard Lina, Oana Dumitrescu

**Affiliations:** 1 Laboratoire des Mycobactéries, Hospices Civils de Lyon, Hôpital de la Croix-Rousse, Centre de Biologie Nord, Institut des Agents Infectieux, 69004 Lyon, France; charlotte.genestet@gmail.com (C.G.); thomas.delque@etu.univ-lyon1.fr (T.D.); luna.ruffel@etu.univ-lyon1.fr (L.R.); yvonne.benito@chu-lyon.fr (Y.B.); isabelle.fredenucci@chu-lyon.fr (I.F.); jean-philippe.rasigade@chu-lyon.fr (J.-P.R.); gerard.lina@chu-lyon.fr (G.L.); oana.dumitrescu@chu-lyon.fr (O.D.); 2 Centre International de Recherche en Infectiologie (CIRI), INSERM U1111, CNRS UMR5308, ENS Lyon, Université Lyon 1, 69001 Lyon, France

**Keywords:** tuberculosis diagnosis, *Mycobacterium tuberculosis* complex, Nucleic acid amplification test, MTB/MDR ELITe MGB^®^ Kit, multidrug-resistant tuberculosis

## Abstract

A rapid and reliable diagnostic for tuberculosis, including the detection of both rifampicin (RIF) and isoniazid (INH) resistance, is essential for appropriate patient care. Nucleic acid amplification tests are a fast alternative to methods based on *Mycobacterium tuberculosis* complex (MTB) cultures. Thus, the performance of the MDR/MTB ELITe MGB^®^ Kit on the ELITe InGenius^®^ platform was retrospectively evaluated for MTB detection on pulmonary and extra-pulmonary samples and for RIF/INH resistance detection on MTB strains. The sensitivity and specificity of the kit for MTB detection compared to the MTB culture were 80.0% and 100.0%, respectively. For the antimicrobial susceptibility prediction, the agreement with phenotypic antimicrobial susceptibility testing (AST) was 92.0%. For RIF, the sensitivity was 100.0% and the specificity was 95.5%. For INH, the sensitivity and specificity were 75.0% and 100.0%, respectively. A single RIF false-positive result was obtained for a strain with a low level of RIF resistance that was not detected by phenotypic AST, but carrying a *rpoB* L452P mutation. INH false-negative results (3) were due to mutations on the *katG* gene that were not probed by the test. Overall, the MTB/MDR ELITe MGB^®^ Kit presents a strong performance for MTB detection and for the detection of both RIF and INH resistance, with an easy integration in laboratory workflow thanks to its fully automatized system.

## 1. Introduction

Tuberculosis (TB) caused by the *Mycobacterium tuberculosis* complex (MTB) remains a worldwide public health concern, including in high-resource countries where TB prevalence is low [1]. The microscopic observation of smears and bacilli cultures from clinical samples is still the recommended method for TB diagnosis, though cultures may be delayed for paucibacillary, smear-negative patients. Moreover, a MTB culture is essential to perform phenotypic antimicrobial sensitivity testing (AST) and thus requires high-level biosafety laboratories [2]. To circumvent these pitfalls, the Center for Disease Control and Prevention recommend using at least one molecular technique per patient for MTB detection, especially for paucibacillary samples, as nucleic acid amplification tests (NAAT) allow a more rapid confirmation of TB diagnosis compared to cultures [2,3]. Moreover, the emergence of multidrug-resistant MTB (MDR-MTB; MTB resistant to isoniazid (INH) and rifampicin (RIF)) remains a challenge for TB management because of the complexity of the treatment in terms of drug associations, side effects, and duration (18–24 months) [4]. Therefore, there is an urgent need to obtain fast and accurate AST results in order to rule out MDR-MTB and rapidly and safely initiate the standard quadritherapy (INH, RIF, pyrazinamide, and ethambutol) [1]. 

As an alternative to the phenotypic and time-consuming AST, the use of rapid molecular tests, such as the Xpert MTB/RIF Ultra^®^ assay (Cepheid, Sunnyvale, CA, USA), which are performed directly on samples and able to detect the most common mutations that confer resistance to RIF, is recommended by the World Health Organization [5]. However, these tests are not able to detect the mutations conferring resistance to INH, yet INH resistance is encountered in 5% to 15% of RIF-susceptible cases worldwide and has a significant impact on treatment outcome [6]. Moreover, the Xpert assay is expensive, even for laboratories in high-resource countries. Alternatively, molecular tests based on line probe assays (LPA) are able to detect the most frequent mutations conferring resistance to RIF and INH and can be performed on MTB strains [2,7]. Nevertheless, LPA require experienced laboratory technicians for the manual processing steps. 

The InGenius^®^ platform is a fully automated sample-to-result PCR system that integrates nucleic acid extraction and real-time PCR amplification and reports directly from primary patient samples in either single analyte or multiplex format. The aim of the present study was to assess the performance of a new automatized NAAT, MDR/MTB ELITe MGB^®^ Kit (ELITechGroup SpA, Torino, Italy) on the ELITe InGenius^®^ platform (ELITechGroup SpA) for pulmonary and extra-pulmonary TB diagnosis and for the detection of mutations conferring resistance to RIF and INH on samples and on MTB strains. 

## 2. Results

### 2.1. Sample Selection

The study took place in the *Laboratoire des Mycobactéries*, *Hospices Civils de Lyon* (Lyon University Hospital Mycobacteria Laboratory) from the French Rhône-Alpes-Auvergne area, which has low TB incidence (7 cases per 1,000,000 inhabitants) and a low proportion of drug-resistant MTB (<0.5%) [8]. The smear-negative samples (pulmonary or extra-pulmonary) containing a low bacterial load, and for which the growth delay in the Mycobacteria Growth Indicator Tube (MGIT) of the MTB culture was prolonged, were purposely selected to challenge the sensitivity of the MTB/MDR ELITe MGB^®^ Kit.

A total of 54 samples were selected before antibiotic treatment: 11 pulmonary smear-positive samples for which the MTB culture was positive (10 sputa, 1 gastric aspirate) containing one RIF-resistant strain, four INH-resistant strains (three low-level resistance and one high-level resistance), and one MDR-MTB strain (RIF-resistant and high-level INH-resistant); 27 pulmonary smear-negative samples for which the MTB culture was positive (11 bronchial aspirates, 10 sputa, 4 broncho-alveolar lavages, 2 gastric aspirates); nine smear-negative extra-pulmonary samples for which the MTB culture was positive (three lymph nodes, three tissue biopsies, two ascites fluids, one psoas abscess); and seven pulmonary samples for which the MTB culture was negative and either the non-tuberculous mycobacteria or *Nocardia* culture was positive (five bronchial aspirates positive for *Mycobacterium avium* (2), *Mycobacterium chimaera* (2) or *Nocardia cyriacygeorgica,* and two sputa positive for *Mycobacterium abscessus*). 

All the samples were tested for the presence of MTB DNA with the MTB/MDR ELITe MGB^®^ Kit: 29 with the ELITe InGenius^®^ SP200 extraction kit and 25 with the ELITe InGenius^®^ SP1000 extraction kit. Invalid results, as a result of the failure in internal control amplification, after two MTB DNA detection tests were obtained for two samples (one bronchial aspirate sample and one lymph node smear-negative sample with a MTB-positive culture) and were excluded from the study.

**Figure 1:**
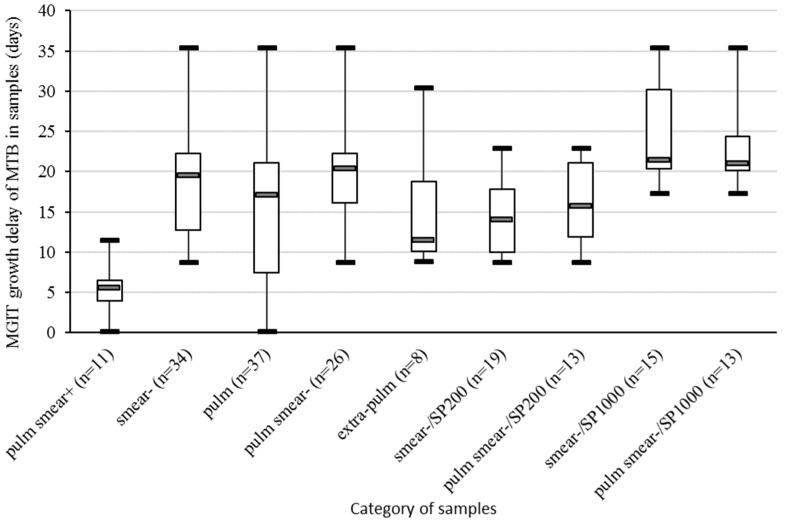
Boxplot representing the MGIT growth delay of the MTB culture in samples included in the study, stratified by smear result (+ or −), type of sample (pulm or extra-pulm), and type of extraction kit (SP200 or SP1000). Pulm, pulmonary samples; extra-pulm, extra-pulmonary samples; MGIT, Mycobacteria Growth Indicator Tubes; MTB, *Mycobacterium tuberculosis* complex.

The MGIT growth delay of the included samples was recorded and stratified according their types, smear results, and type of extraction (Figure 1). 

### 2.2. MTB Strains Selection

**Figure 2:**
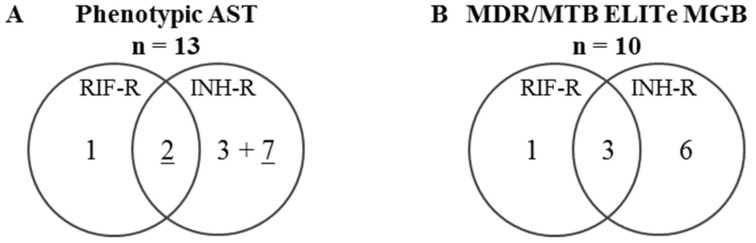
Venn diagram of the distribution of MTB strains depending on their resistance against RIF and INH. The resistance was determined (**A**) using phenotypic AST or (**B**) by a genotypic analysis test using the MTB/MDR ELITe MGB^®^ Kit. R: resistant, RIF: rifampicin, INH: isoniazid, AST: antimicrobial susceptibility testing.For INH-R, underlined numbers correspond to MTB isolates with a high-level of INH resistance (determined by INH resistance at CC 0.1 and 0.4 mg/L for phenotypic AST).

A total of 25 MTB strains were selected, including 13 isolates (52%) resistant to either RIF and/or INH according to phenotypic AST (Figure 2A), and previously characterized by LPA and whole-genome sequencing (WGS) [9]. 

### 2.3. Performance of the MDR/MTB ELITe MGB^®^ Kit for MTB DNA Detection on Samples

**Figure 3:**
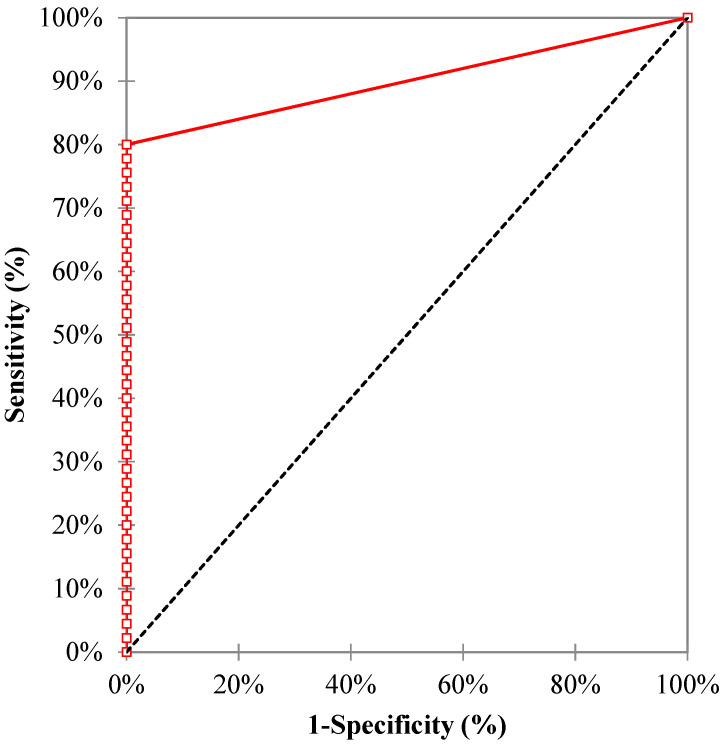
The ROC curve of the MDR/MTB ELITe MGB^®^ Kit for the diagnosis of tuberculosis.

The sensitivity, specificity, positive predictive value (PPV), and negative predictive value (NPV) of the MDR/MTB ELITe MGB^®^ Kit were 80.0% (95% confidence interval, CI95 (65.4; 90.4)), 100.0% (CI95 (59.0; 100.0)), 100.0% (CI95 (90.3; 100.0)), and 43.8% (CI95 (19.8; 70.1)), respectively. The area under the ROC (receiver operating characteristic) curve (Figure 3) was 0.90 (CI95 (0.84; 0.96)), with a *p* value < 0.0001.

**Figure 4:**
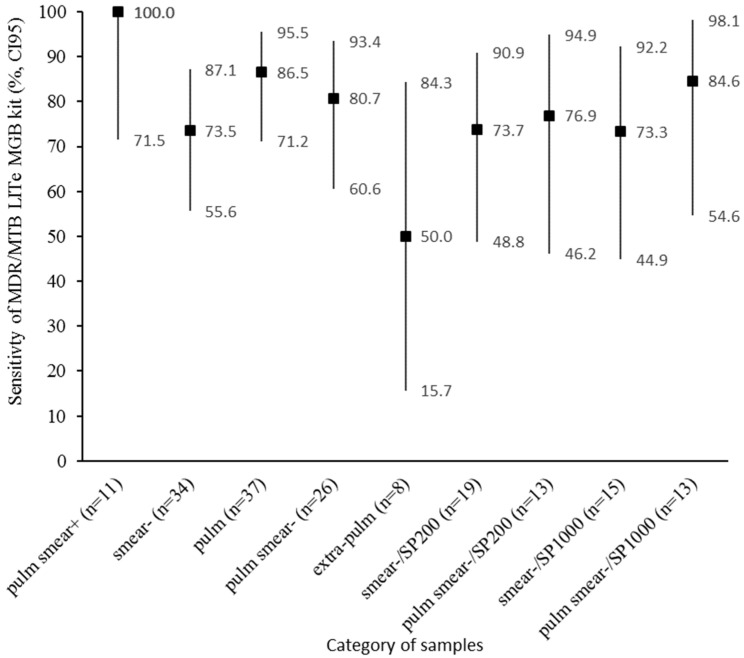
Sensitivity of the MDR/MTB ELITe MGB^®^ Kit for MTB DNA detection, stratified by smear result (+ or −), type of sample (pulm or extra-pulm), and type of extraction kit (SP200 or SP1000). Pulm, pulmonary samples; extra-pulm, extra-pulmonary samples; MGIT, Mycobacteria Growth Indicator Tubes; MTB, *Mycobacterium tuberculosis* complex; CI95, 95% confidence interval.

The sensitivity was also evaluated after stratification by smear result, type of sample, and type of extraction kit (Figure 4).

**Figure 5:**
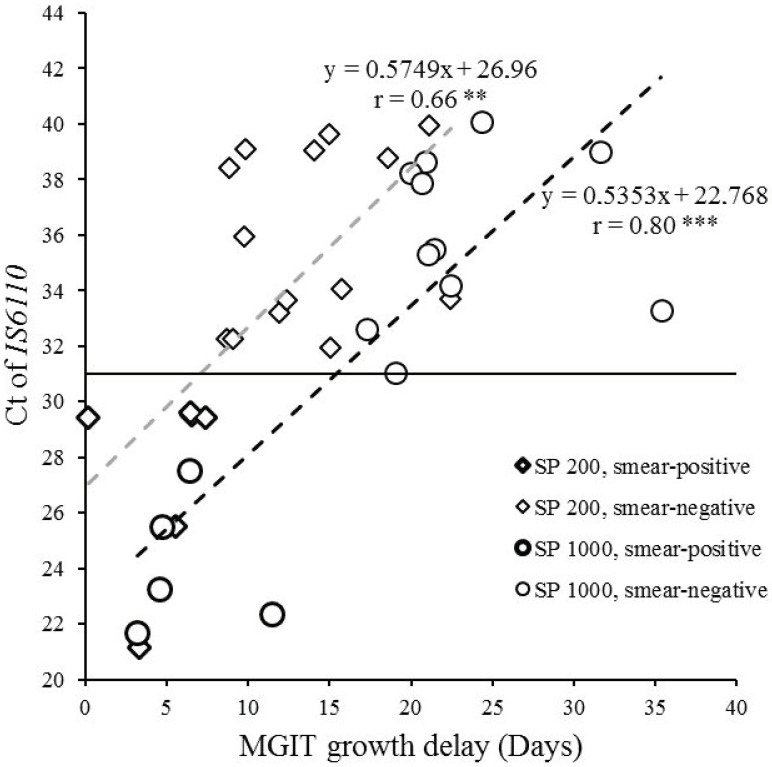
Representation of the MGIT growth delay according to the quantitative measurement of MTB DNA (*IS6110* Ct) in specimens. Diamonds indicate specimens processed with the ELITe InGenius^®^ SP200 extraction kit (n = 20, including six smear-positive samples in bold outline); circles indicate specimens processed with the ELITe InGenius^®^ SP1000 extraction kit (n = 16, including five smear-positive samples in bold outline). Pearson’s correlation test, ** *p* < 0.01, *** *p* < 0.001. Ct, cycle threshold.

The time of growth was significantly positively correlated with the values of the cycle threshold (Ct) of *IS6110* for the samples processed with the ELITe InGenius^®^ SP200 extraction kit (r = 0.66, CI95 (0.30; 0.85), *p* < 0.01) and the SP1000 extraction kit (r = 0.80, CI95 (0.51; 0.93), *p* < 0.001), as shown in Figure 5. The slope of both correlation lines was quite similar (about 0.5), the intercept was 22.768 for SP1000 and 26.96 for SP200, suggesting that the samples tested with the SP1000 (1 mL) could be approximately 4 Ct lower than samples tested with the SP200 (200 µL).

### 2.4. Performance of the MDR/MTB ELITe MGB^®^ Kit for MTB Resistance Detection on Samples

Among the 36 samples that tested positive for MTB DNA detection (32 pulmonary and 4 extra-pulmonary samples), 11 samples (30.6%) displayed an *IS6110* Ct ≤ 31, allowing MTB resistance detection (Figure 3). All them were smear-positive pulmonary samples. 

Among the 11 smear-positive samples, 6 samples containing a MTB strain phenotypically characterized as resistant to RIF and/or INH were tested using the MDR/MTB ELITe MGB^®^ Kit for MTB resistance detection, and a concordance of 100.0% (12/12) (CI95 (73.5; 100.0)) between the prediction of the kit and phenotypic AST determination was obtained.

### 2.5. Performance of the MDR/MTB ELITe MGB^®^ Kkit for MTB Resistance Detection on MTB Strains

The performance of the MTB/MDR ELITe MGB Kit for the prediction of RIF or INH resistance or susceptibility on MTB strains is compared to phenotypic AST and reported in Table 1.

A total of 10 MTB strains were predicted as resistant to either RIF and/or INH using the MTB/MDR ELITe MGB^®^ Kit (Figure 2B). For these 10 MTB strains, the melting temperature (Tm) of the MDR/MTB ELITe MGB altered probe was analyzed and compared with the WGS result (Table 2). 

### 2.6. Discrepancy Analysis

Among the 50 antimicrobial statue predictions obtained using the MTB/MDR ELITe MGB^®^ Kit on MTB strains, 46 were concordant with phenotypic AST, and the overall agreement was 92.0% (46/50; CI95 (80.8; 97.8)). The concordance testing showed a high concordance between phenotypic AST and genotypic AST using the MTB/MDR ELITe MGB^®^ Kit: Cohen’s kappa was 0.757 and 0.834 for INH and RIF, respectively.

The discrepancies between the phenotypic AST and the MTB/MDR ELITe MGB^®^ Kit regarded resistance against RIF in one-fourth of the cases and against INH in three-fourths of the cases. For these strains, LPA and WGS were also recorded and analyzed (Table 3).

## 3. Discussion

The overall sensitivity and specificity of the MTB/MDR ELITe MGB^®^ Kit reported in this study were very good, and the ROC curve analysis indicated a high accuracy of the test for TB diagnosis. Nevertheless, the overall sensitivity and the sensitivity on pulmonary samples were slightly inferior compared to these reported by Bisognin et al. (90.8%; CI95 (84.6; 94.6) and 98.0%; CI95 (89.3; 99.9), respectively) [10]. This discrepancy is probably due to the way the samples were selected, as the MTB inoculum was higher in the latter study: a majority of the pulmonary samples were smear-positive (44/50), and when MTB was detected, the Ct of *IS6110* was ≤31 (molecular typing feasible) for 95.5% of the pulmonary samples and 46.2% of the extra-pulmonary samples. A similar sensitivity was previously reported (81.8%; CI95 (64.5; 93.0)) on smear-negative pulmonary samples using the Xpert MTB/RIF Ultra (recommended by WHO) [11]. 

The sensitivity of the MTB/MDR ELITe MGB^®^ Kit on the extra-pulmonary samples observed in this study was moderate, and lower than the sensitivity reported by Bisognin et al. (86.3%; CI95 (76.7; 92.9)) [10]. Using the Xpert MTB/RIF Ultra, a recent meta-analysis found a much higher sensitivity (85.6%; CI95 (76.7; 91.5)) on extra-pulmonary samples [12], suggesting that the latter test could be more sensitive on this type of sample than the MTB/MDR ELITe MGB^®^ Kit. Nevertheless, the present study included only a few extra-pulmonary samples, purposefully selected to contain a very small amount of MTB, which may explain the low sensitivity on extra-pulmonary samples. 

Using the SP1000 extraction kit, the sensitivity was similar or slightly superior to the SP200 extraction kit, although the MGIT growth delay was longer for the SP1000-extracted samples compared to the SP200-extracted samples. Overall, the samples tested with the SP1000 extraction kit were approximately 4 Ct lower than the samples tested the SP200 extraction kit, thus using the SP1000 extraction kit could increase the sensitivity of the test. Further studies comparing the Ct of *IS6110* on paired samples would be helpful to confirm this point. 

The performance of the MTB/MDR ELITe MGB^®^ Kit for the detection of RIF and INH resistance was rather good. The agreement between the predictions of the kit and the phenotypic AST on the samples was perfect, but only a few samples were analyzed. The CI95 around the point estimates of sensitivity and specificity on MTB strains was important, because the number of MTB isolates included in the present study was low. Nevertheless, the RIF resistance prediction was very good (only one false-positive result). For this particular strain, WGS found a *rpoB* L452P mutation that was previously reported to be associated with low-level resistance to rifampicin, and the strain was found to be susceptible by phenotypic AST (MGIT system), but associated with relapse or treatment failure [13]. In such cases, RIF resistance prediction using the MTB/MDR ELITe MGB^®^ Kit could be beneficial for the patient, as it would prevent the need to use the usual dosage of RIF to treat TB and avoid the associated risk of relapse. Importantly, LPA failed to predict the RIF resistance for this strain. For INH susceptibility prediction on MTB strains, the sensitivity of the MTB/MDR ELITe MGB^®^ Kit was lower because of the non-detection of some mutations not probed by the method (*katG Δ1-492*, *katG Q88P*, *katG L343STOP*), leading to false INH susceptibility results. Contrary to the WGS, the targeted genotypic methods, such as the MTB/MDR ELITe MGB^®^ Kit, only analyze small sequences of the genome carrying the main mutations that induce INH resistance (Table 4). These defects were also observed using the LPA. On the other hand, the specificity of the MTB/MDR ELITe MGB^®^ Kit was excellent for indicating the absence of false-positive results for INH. The LPA was found to have a similar performance [9]. Interestingly, using the MTB/MDR ELITe MGB^®^ Kit, a different Tm for each detected mutation in WGS was observed. Therefore, the Tm shift does not only indicate the resistance status, but also could be used to predict the precise mutation responsible for antimicrobial resistance in the MTB isolate. 

The integration of the MTB/MDR ELITe MGB^®^ Kit (consumable reagent cost: 21.6 euros per test for TB1+TB2 PCR; 18.5 euros per test for TB1 screening) into the laboratory workflow is a fully automatized method, but it requires the ELITe InGenius^®^ platform, the acquisition of which requires substantial means. Compared to the Xpert MTB/RIF Ultra (consumable reagent cost: 54.0 euros per test), which also requires a specific platform and that processes samples one-by-one, the consumable reagent cost of the MTB/MDR ELITe MGB^®^ Kit is less expansive: the MTB/MDR ELITe MGB^®^ Kit can process samples (single or multi-parameter) serially (maximum 12 samples per run for TB1 screening and 6 samples per run for TB1 + TB2 analysis), and additionally allows for the testing of INH genotypic susceptibility. Notably, ELITe InGenius^®^ works as part of a dedicated molecular diagnosis platform, and contrary to Xpert, ELITe InGenius^®^ requires heat-inactivation of the samples prior to analysis. Compared to LPA, which does not require a specific platform (consumable reagent cost: 23.2 euros per test), the consumable reagent cost of the MTB/MDR ELITe MGB^®^ Kit is similar, but allows for a better laboratory efficiency through automation. 

## 4. Materials and Methods

### 4.1. Samples and MTB Strain Selection

Respiratory and extra-respiratory samples and MTB strains were sampled from patients during routine care, between March 2017 and April 2020 for samples, and between April 2016 and July 2020 for MTB strains, in the *Laboratoire des Mycobactéries* of the *Hospices Civils de Lyon* in France. These samples were retrospectively selected and analyzed. Smear-negative samples collected before antimicrobial treatment, containing a low bacterial load, and with a prolonged growth time in the MGIT culture, were purposely selected to challenge the sensitivity of the MTB/MDR ELITe MGB^®^ Kit. The number and the type of sample collected were consistent with the requirements for method validation in the accreditation process of the clinical laboratory. This study was declared to the ethics committee of the *Hospices Civils de Lyon* in France (declared sample collection: DC-2011-1306). In accordance with French legislation, written informed consent from patients was not required.

### 4.2. Culture of MTB

After the treatment of the pulmonary samples with the modified Kubica’s digestion- decontamination method (N-acetyl-L-cysteine–2% NaOH (sodium hydroxide)), the samples were centrifuged and the pellets were re-suspended in a final volume of 2 mL [14]. Smear staining was performed using the acridine orange method (one smear observed, performed with 50 μL of the sample) [15]. MTB cultures were performed by the inoculation of 500 μL of the re-suspended sample in the MGIT using the BACTEC 960^®^ instrument (Becton Dickinson, Sparks, MD, USA), by the inoculation of 200 µL of the re-suspended sample in the Coletsos medium (Bio-Rad, Hercules, CA, USA), and the incubation of the cultures for 90 days at 37 °C. The MGIT growth delay was recorded for each sample. The remaining sample was heat-inactivated (at 95 °C for 15 min) and stored at −20 °C.

### 4.3. Nucleic Acid Amplification Tests on Samples

The samples were tested with the MDR/MTB ELITe MGB^®^ Kit with the ELITe InGenius^®^ SP200 (processing 200 mL of the sample) or SP1000 (processing 1 mL of the sample) extraction kit on the ELITe InGenius^®^ platform, according to the manufacturer’s recommendations. To evaluate the performance of the MDR/MTB ELITe MGB^®^ Kit for MTB DNA detection, only TB1 (targeting repeated sequence *IS6110*, and containing 3 of the 4 probes targeting the *rpoB* gene: rpoB2, rpoB3, and rpoB4) PCR mix reagents were used, while to evaluate the performance for MTB resistance detection, both TB1 and TB2 (targeting the *rpoB* gene with the rpoB1 probe, the *katG* with the katG probe, and the promoter of *inhA* with the inhA probe) PCR mix reagents were used. To obtain a well-controlled and interpretable result regarding MTB resistance by the analysis of melting temperature (Tm) curves of different probes, the samples should contain enough MTB DNA, i.e., the Ct for *IS6110* should be ≤31. The mutations evaluated by the kit are listed in Table 4.

### 4.4. Genotyping of MTB Strains 

Genotyping of the MTB-positive culture was performed by LPA using the GenoType MTBDR*plus* v.2.0 test (Hain Lifescience GmbH, Nehren, Germany) according to the manufacturer’s instructions, and by whole-genome sequencing (WGS), as described elsewhere [9]. Genotyping of the MTB-positive culture was also performed using the MDR/MTB ELITe MGB^®^ Kit (mix TB1 and TB2) with the ELITe InGenius^®^ SP200 on the ELITe InGenius^®^ platform. For the MTB strains cultured in the Coletsos medium, colonies were suspended in 2 mL of physiologic water (*bioMérieux*, Marcy-l’Etoile, France), heat-inactivated at 95 °C for 15 min, and diluted at 1:1000 in physiological water. For the MTB strain cultures in the liquid medium, a sample of 2 mL was collected from the MGIT, heat-inactivated, and diluted at 1:100 in physiological water.

### 4.5. Phenotypic Antimicrobial Susceptibility Testing

Phenotypic AST using the MGIT AST SIRE system and the BACTEC 960^®^ instrument (Becton Dickinson) was performed for RIF (critical concentration (CC) 1.0 mg/L) and INH (CC 0.1 mg/L and 0.4 mg/L), according to the manufacturer’s instructions [16]. INH resistance was considered to be low when the strain was resistant at a CC of 0.1 mg/L and susceptible at a CC of 0.4 mg/L, and high when the strain was resistant at a CC of 0.4mg/L. For 1 MDR-MTB strain, the reference proportion method on Löwenstein–Jensen medium for AST was performed at the French National Reference Center (NRC) for Mycobacteria and anti-tuberculous drug resistance.

### 4.6. Whole-Genome Sequencing

Whole-genome sequencing of all isolates was performed as described elsewhere [15]. The sequences were submitted to European Nucleotide Archive (ENA) under the accession number PRJEB42621. 

### 4.7. Data Analysis

For MTB DNA detection in samples, the sensitivity, specificity, positive predictive value (PPV), and negative predictive value (NPV) were calculated using the MTB-positive culture as the standard. Pearson’s correlation test was performed to test the correlation between the MGIT growth delay and the quantitative measurement of MTB DNA, given by the Ct of *IS6110*. For MTB resistance detection, phenotypic AST was used as a reference to calculate the sensitivity (prediction of antibiotic resistance), specificity (prediction of antibiotic susceptibility), PPV, and NPV of the MDR/MTB ELITe MGB^®^ Kit. 

Statistical analyses were performed using RStudio, version 0.99.893 (RStudio Team (2009–2016), RStudio: Integrated Development for R. RStudio, Inc., Boston, MA, USA). The ROC curve analyses and Cohen’s kappa were performed using XLSTAT 2020.5.1. 

## 5. Conclusions

**Figure 6:**
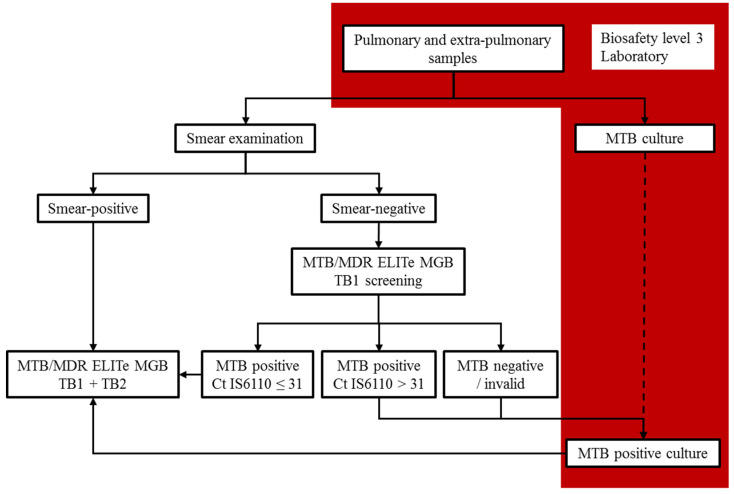
Integration of the MTB/MDR ELITe MGB^®^ Kit in laboratory workflow.

In conclusion, MTB/MDR ELITe MGB^®^ is an automatized system that has very good performance for MTB detection, including in paucibacillary samples, and especially in smear-negative pulmonary samples, but it requires a dedicated molecular diagnosis platform. For optimal sensitivity, the use of the SP1000 extraction kit should be favored. The detection of both RIF and INH resistance using the MTB/MDR ELITe MGB^®^ Kit is feasible on MTB strains with very good performance, similar to LPA, but it is easier to implement in laboratory workflow through automation. Because the number of tested samples was low in this study, additional data are needed to consolidate these conclusions. Thus, we suggest an efficient laboratory workflow that integrates this test in TB diagnosis, and is compatible with all clinical laboratories that have a high-level biosafety facility (Figure 6).

## Figures and Tables

**Table 1:** The performance of MTB/MDR ELITe MGB Kit for the prediction of the susceptibility profile of *Mycobacterium tuberculosis* isolates to isoniazid and rifampicin, considering phenotypic antimicrobial susceptibility testing as the reference.

Antibiotic	Number of Isolates Phenotypically		Sensitivity CI95	Specificity CI95	PPV CI95	NPV CI95
	Resistant	Susceptible				
RIF	3	22	100.0 [29.2; 100.0]	95.5 [77.2; 99.9]	75.0 [19.4; 99.4]	100.0 [83.9; 100.0]
INH	12	13	75.0 [42.8; 94.5]	100.0 [75.3; 100.0]	100.0 [66.4; 100.0]	81.3 [54.4; 95.6]

CI, confidence interval; RIF, rifampicin; INH, isoniazid; PPV, positive predictive value; NPV, negative predictive value.

**Table 2:** Analysis of MDR/MTB ELITe MGB altered probe Tm for the 10 RIF- or INH-resistant MTB strains.

Genotypic AST Prediction	MDR/MTB ELITe MGB Kit Result			WGS Result
	Altered Probe	Tm Limits (°C)	Tm (°C) of the Altered Probe	
**RIF-R**	rpoB2	70.0–80.0	68.9	*rpoB* L452P
			57.3 57.2	*rpoB* S450L
	rpoB3	68.0–80.0	N.D (not amplified)	*rpoB* H445Y
**INH-R**	katG	69.0–80.0	66.1 66.4 66.6 (2 strains) 66.4 (2 strains)	*katG* S315T
	inhA	66.0–80.0	63.5	-17*fabG1*
			59.3 58.8	-15*fabG1*

AST, antimicrobial susceptibility testing; RIF, rifampicin; INH, isoniazid; R, resistance; Tm, melting temperature; WGS, whole-genome sequencing; LPA, line probe assay.

**Table 3:** Discrepancies between the phenotypic and genotypic AST (WGS, MTB/MDR ELITe MGB Kit, LPA).

Strain Number	Phenotypic AST	Genotypic AST Prediction		
		WGS	MTB/MDR ELITe MGB Kit	LPA
0170529571	RIF-susceptible	RIF-resistant *rpoB* L452P	RIF-resistant *rpoB* mutation detected (rpoB2 probe)	RIF-susceptible *rpoB* mutation not detected
0160712581	INH-resistant with high level	INH-resistant *katG* Δ1-492	INH-susceptible *katG* mutation not detected	INH-susceptible *katG* mutation not detected
0162370229	INH-resistant with low level	INH-resistant *katG* Q88P	INH-susceptible *katG* mutation not detected	INH-susceptible *katG* mutation not detected
0170485653	INH-resistant with high level	INH-resistant *katG* L343STOP	INH-susceptible *katG* mutation not detected	Uninterpretable (lack of *katG* locus control band)

RIF, rifampicin; INH, isoniazid; AST, antimicrobial susceptibility testing; WGS, whole-genome sequencing; LPA, line probe assay.

**Table 4:** List of the mutations detected by MDR/MTB ELITe MGB^®^ Kit according the manufacturer’s notice.

Mutations in the 81 bp hot-spot region of the *rpoB* gene (numbering of *E. coli* codons)
Q510L, L511P, L511R, Q513L, Q513P, M515I, D516V, D516Y, D516G, Q517P, S522L, S522P, H526L, H526Y, H526D, H526N, H526R, H526C, H526P, S531L, S531W, A532V, L533P
Mutations in the region of codon 315 of the *katG* gene
S315N, S315T
Mutations in the promoter region of the *inhA* gene
-15T, -8A, -8C, -7A

## Data Availability

The sequences were submitted to European Nucleotide Archive (ENA) under the accession number PRJEB42621.
